# Effects of a swallowing and oral-care program on resuming oral feeding and reducing pneumonia in patients following endotracheal extubation: a randomized, open-label, controlled trial

**DOI:** 10.1186/s13054-023-04568-6

**Published:** 2023-07-12

**Authors:** Shu-Fen Siao, Shih-Chi Ku, Wen-Hsuan Tseng, Yu-Chung Wei, Yu-Chun Chang, Tzu-Yu Hsiao, Tyng-Guey Wang, Cheryl Chia-Hui Chen

**Affiliations:** 1 School of Nursing, National Taiwan University College of Medicine, 1, Jen-Ai Road, Section 1, Taipei, 100 Taiwan; 2 Department of Internal Medicine, National Taiwan University Hospital and National Taiwan University College of Medicine, Taipei, Taiwan; 3 Department of Otolaryngology, National Taiwan University Hospital and National Taiwan University College of Medicine, Taipei, Taiwan; 4 Graduate Institute of Statistics and Information Science, National Changhua University of Education, Changhua, Taiwan; 5 Department of Physical Medicine and Rehabilitation, National Taiwan University Hospital and National Taiwan University College of Medicine, Taipei, Taiwan; 6 Department of Nursing, National Taiwan University Hospital and National Taiwan University College of Medicine, Taipei, Taiwan

**Keywords:** Endotracheal intubation, Dysphagia, Oral feeding, Swallowing, Mechanical ventilation, Extubation, Nurses

## Abstract

**Background:**

The resumption of oral feeding and free from pneumonia are important therapeutic goals for critically ill patients who have been successfully extubated after prolonged (≥ 48 h) endotracheal intubation. We aimed to examine whether a swallowing and oral-care (SOC) program provided to critically ill patients extubated from prolonged mechanical ventilation improves their oral-feeding resumption and reduces 30-day pneumonia incidence.

**Methods:**

In this randomized, open-label, controlled trial, participants were consecutively enrolled and randomized to receive the SOC program or usual care. The interventions comprised three protocols: oral-motor exercise, sensory stimulation and lubrication, and safe-swallowing education. Beginning on the day following patient extubation, an SOC nurse provided the three-protocol care for seven consecutive days or until death or hospital discharge. With independent outcome assessors, oral-feeding resumption (yes, no) corresponded to level 6 or level 7 on the Functional Oral Intake Scale (censored seven days postextubation) along with radiographically documented pneumonia (yes, no; censored 30 days postextubation), abstracted from participants’ electronic medical records were coded.

**Results:**

We analyzed 145 randomized participants (SOC group = 72, control group = 73). The SOC group received, on average, 6.2 days of intervention (14.8 min daily) with no reported adverse events. By day 7, 37/72 (51.4%) of the SOC participants had resumed oral feeding vs. 24/73 (32.9%) of the control participants. Pneumonia occurred in 11/72 (15.3%) of the SOC participants and in 26/73 (35.6%) of the control participants. Independent of age and intubation longer than 6 days, SOC participants were likelier than their control counterparts to resume oral feeding (adjusted hazard ratio, 2.35; 95% CI 1.38–4.01) and had lower odds of developing pneumonia (adjusted odds ratio, 0.28; 95% CI 0.12–0.65).

**Conclusions:**

The SOC program effectively improved patients’ odds that oral feeding would resume and the 30-day pneumonia incidence would decline. The program might advance dysphagia care provided to critically ill patients extubated from prolonged mechanical ventilation.

*Trial registration*: NCT03284892, registered on September 15, 2017.

**Supplementary Information:**

The online version contains supplementary material available at 10.1186/s13054-023-04568-6.

## Background

The resumption of oral feeding and free from pneumonia are important therapeutic goals for critically ill patients who have been successfully extubated after prolonged (≥ 48 h) endotracheal intubation [1, 2]. Up to 41% of intensive-care unit (ICU) patients with prolonged endotracheal intubation experienced postextubation dysphagia, as they had difficulty resuming oral feeding [3, 4], leading to increased risk of pneumonia [5–7] and higher 90-day mortality [8].

Although these consequences of post extubation dysphagia are commonly observed, few studies have examined interventions to resolve the problems. For decades, researchers have studied the theoretical scope of swallowing rehabilitation with respect to oropharyngeal exercises [9–12], oral hygiene and moisturization [13–16], and such compensatory strategies as changes in posture and food viscosity [16–18]. However, there is limited research on the bedside application of swallowing rehabilitation (i.e., care protocols), especially in critically ill patients receiving prolonged mechanical ventilation. Previously, we bundled these rehabilitation approaches and carefully translated them into a nurse-administered, 14-day swallowing and oral-care (SOC) intervention. As demonstrated in a pre- and post-intervention pilot study, this 14-day SOC intervention provided to ICU patients after extubation was linked with improvements in patients’ salivary-flow rates and a 1.77-fold increase in their odds of resuming oral feeding [19]. The present study was a follow-up of our pilot. As an open-label, randomized controlled trial (RCT), we modified the SOC intervention by adding sour-flavor ice pops and pork jerky to generate thermal-tactile oral stimulation [20–22] that is feasible for consistent bedside delivery. We also shortened the duration of the SOC intervention from 14 to 7 days to minimize the care burden so the clinical application could be maximized. Thus, the purpose of this RCT was to evaluate the effects of this modified, 7-day, nurse-administered SOC program on the resumption of oral feeding and the incidence of radiographically documented pneumonia in consecutively enrolled adult (≥ 20 years old) critically ill patients who received prolonged endotracheal intubation and have been successfully extubated. Amount of time spent delivering the intervention, patients’ adherence to the SOC regimen, and adverse events during and right after the intervention were also evaluated.

## Methods

### Study design

This RCT registered at the Clinical Trials Registry (Trial No. NCT03284892) and was approved by the Human Research Ethics Committee at National Taiwan University Hospital (201705051RIND). All the procedures were followed in accordance with the ethical standards on human experimentation and with the Helsinki Declaration of 1975 and its later amendments. Written informed consent was obtained from all participants.

### Participants and settings

This trial was conducted at six medical ICUs at a tertiary medical center in Taipei, Taiwan. Consecutive patients (≥ 20 years old) were recruited from September 2017 to July 2020 if they had received emergency oral endotracheal intubation for at least 48 h and had been successfully extubated. Patients were excluded if they (1) had a history of neuromuscular disease (e.g., parkinsonism or stroke) or head and neck deformities, (2) had preexisting difficulty swallowing, (3) had received a tracheostomy, (4) were unable to follow verbal instructions, (5) were on contact and droplet precautions (e.g., open tuberculosis), or (6) were receiving continuous noninvasive ventilation after extubation that precluded the delivery of an SOC intervention.

### Randomization and blinding

Based on a computer-generated random sequence, participants were randomly assigned, on a 1:1 ratio, to an intervention, SOC group or a control group (receiving usual care). To maintain allocation concealment, we ensured that only the intervention nurse had access to the random sequence. Physicians and staff at the study sites were aware of a pending nursing-intervention study but were blinded with respect to the hypothesis, group allocation, specific SOC protocols, and study endpoints. Moreover, outcome assessors were blinded to the group assignments, ensuring an unbiased evaluation.

### SOC program and usual care

Participants in the SOC group, in addition to the usual care, received daily SOC for seven days or until death or hospital discharge (whichever occurred first) starting on the day following extubation (regardless of intake status). The SOC consisted of three protocols: oral motor exercise (exercises for the lips, tongue, jaw, and cheeks), sensory stimulation and lubrication (thermal-tactile stimulation, toothbrushing, and salivary-gland massage), and safe-swallowing education (Additional file 1). The SOC nurse tracked all interventions daily and rated the participants’ adherence to the regimen on a Likert-type scale ranging from 0 (no or limited adherence) to 2 (full adherence). Neither the physician nor the medical staff had access to the details of the SOC intervention, and the SOC nurse had no contact with the medical team. The SOC was delivered by two trained nurses with significant ICU and rehabilitation work experience; they received two-month training, provided by a multidisciplinary team, including experienced research nurses, a physician specialized in rehabilitative medicine, and a speech-language pathologist (SLP). This training prepared them to effectively implement the SOC program in the ICU setting. The fidelity of the SOC was maintained through regular meetings with quality control activities including review of intervention logs to ensure the SOC nurses' competence in executing the SOC program and to address any questions or concerns during the study period.

Usual care consisted of daily oral care, provided each shift by ICU nurses using oral swabs and rinsing with 2% chlorhexidine gluconate. A rehabilitation doctor or SLP provided additional care only at the attending physician’s request. No standardized oral or swallowing care was routinely provided for post-extubated patients after their ward transfer.

### Data collection and outcome measures

Participant characteristics abstracted from medical records included age, sex, body mass index, Charlson Comorbidity Index, ICU admission diagnosis, illness severity (Acute Physiological and Chronic Health Evaluation II [APACHE II]), size of endotracheal tube, and length of intubation (in days). After participants were successfully extubated, their level of consciousness (measured on the Glasgow coma scale), oxygen demand (room air, nasal cannula, simple mask, and nonrebreathing mask [NRM] or noninvasive positive pressure ventilation [NPPV]), functional status on oral stereognosis (able to identify three shapes of lollipop-style test pieses [yes, no]) and cough reflex (able to cough during 0.4 mol/L citric acid inhalation trial, [yes, no]), nothing-by-mouth status (8-h postextubation), and presence of dry mouth were evaluated by outcome assessors. As an important covariate in our previous studies [19, 23], dry mouth (defined as a wetting length of less than 3 cm on a Schirmer’s tear test strip measured 5 min after placement of the strip on the floor of a participant’s mouth) was assessed according to a standardized protocol [24].

### Intervention outcomes

#### Resumption of oral feeding

Outcome assessors used the Functional Oral Intake Scale (FOIS) to assess all participants’ postextubation intake status daily. The FOIS is a valid, 7-level ordinal measure that describes the functional level of a patient’s actual daily oral intake of food and liquid [25]. On the present study, level 6 (total oral diet with multiple consistencies, without special preparation but with specific food limitations) or level 7 (total oral diet with no restriction) indicated that the participants had resumed oral feeding. We compared the two participant groups regarding their resumption of oral feeding seven days following extubation. Notably, the physician decision to resume oral feedings was based on pre-defined criteria, which did not include any information from the SOC intervention.

#### Radiographically documented pneumonia

Two investigators manually abstracted from electronic medical records whether pneumonia was present in participants 30 days postextubation (yes, no). Evidence of a new or progressive radiographically documented infiltrate plus any two of the following four clinical features define pneumonia: (1) fever or hypothermia (body temperature > 38 °C or < 36 °C), (2) leukocytosis or leukopenia (white blood cell count > 11,000 per mm^3^ or < 4000 per mm^3^, respectively), (3) purulent secretions, and (4) a decline in oxygenation [26, 27]. Participants who met the above criteria on at least one occasion during the 30-day observation period were classified into the pneumonia-positive group.

Two investigators who were blinded to the participants’ group adjudicated pneumonia outcomes, with discrepancies resolved within a group discussion involving a third blinded investigator. In our center, chest x-rays and complete blood counts with differential were monitored on a routine basis: weekly or, if pneumonia is suspected, more frequently.

The SOC nurse documented the time spent on providing the SOC program, patients’ adherence to the SOC protocols, and whether any adverse events (coughing, wet voice, respiratory rate > 30 breaths per minute, and decreased oxygen saturation) surfaced during or immediately after the intervention. Adherence to the SOC program or to each individual protocol was rated daily by the SOC nurse and calculated as the mean adherence scores ranging across three main points: 0 (no or limited adherence), 1 (partial adherence), and 2 (full adherence).

### Sample size estimation

We used PASS software version 15.0.5 (NCSS, LLC, Kaysville, UT) to perform sample-size estimations. Based on our pilot findings, we targeted a hazard ratio of 1.77 and estimated that 68% of the intervention group and 54% of the control group would resume total oral intake [19]. We thus calculated that, with a one-sided α of 0.05, an enrollment of 125 patients (62 in the controls and 63 in the treatment group) would provide our study with 80% power for the detection of a similar magnitude of treatment effect. Thus, our target enrollment was set at 140 patients (70 per group).

### Statistical analysis

We performed all analyses based on the intention-to-treat principle. We summarized continuous variables as either means with standard deviations (SDs) or medians with interquartile ranges (IQR); categorical variables were summarized by frequency (percentage). We compared the outcome data between the SOC and controls and reported the proportional differences for categorical variables, with a 95% confidence interval (CI). For the time-to-event analysis, the resumption of oral feeding was plotted by the Kaplan–Meier curve and tested with a log-rank test. We used the Cox proportional hazards model to estimate hazard ratio (HR) with a 95% CI. For pneumonia (yes, no), we used logistic regression to obtain odds ratios (ORs) with a 95% CI. Age and length of intubation are pre-defined covariates based on prior study findings [3, 19, 28] and thus were forced into all models. All analyses were performed with SAS software version 9.4 (SAS Institute Inc., Cary, NC). Significance was set at *P* < 0.05.

## Results

**Fig. 1:**
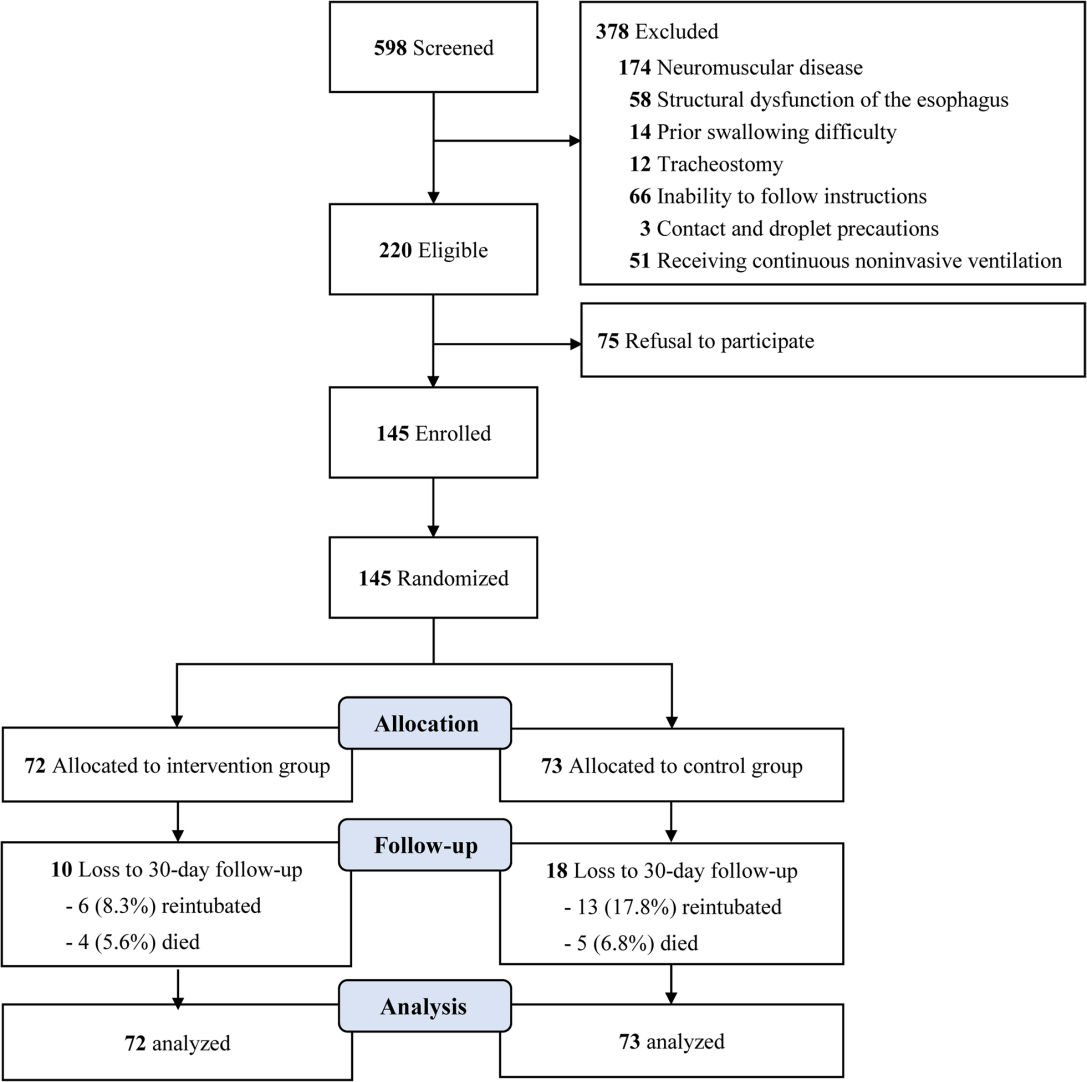
Study flow diagram

As shown in Fig. 1, 145 eligible participants were enrolled and randomly assigned to the SOC group (*n* = 72; mean [SD] age, 67.4 [15.4] years; 41 [56.9%] male) or the control group (*n* = 73; mean [SD] age, 64.2 [14.4] years; 44 [60.3%] male) (Table 1). We noted similarities between the two groups regarding participants’ baseline characteristics, including APACHE II scores at ICU admission (mean [SD], 21.9 [6.8] for the SOC vs. 20.9 [7.6] for the controls), length of intubation (median [IQR], 6.1 [4.1–10.1] days for the SOC vs. 5.8 [4.2–10.2] days for the controls), endotracheal tube sizes, and postextubation oxygen demand. Notably, 28 participants were lost to follow-up due to reintubation (6 in the SOC group and 13 in the control group) or death (4 in the SOC group and 5 in the control group). Participants who were unable to complete 30-day follow-up showed a significantly longer length of intubation (8.8 days vs. 5.4 days) with a higher percentage of intubation over 6 days (78.6% vs. 41%). Additionally, they also presented higher values of rapid shallow breathing index (64.1 vs. 47.8) than those who completed the study (Additional file 2: Table S1).

**Table 1:** Baseline demographics and clinical characteristics of the participants

Variables	SOC (*n* = 72)	Control (*n* = 73)
Age, mean (SD), y	67.4 (15.4)	64.2 (14.4)
Age ≥ 70 y, *n* (%)	36 (50)	27 (36.9)
Male, *n* (%)	41 (56.9)	44 (60.3)
Body mass index, mean (SD)	24.2 (5.1)	22.5 (3.2)
Charlson Comorbidity Index, mean (SD)	2.7 (2.2)	2.8 (2.6)
ICU admission diagnosis, *n* (%)		
Respiratory failure	40 (55.7)	44 (60.2)
Cardiac emergency	14 (19.4)	13 (17.8)
Noncardiogenic shock	14 (19.4)	14 (19.2)
Others^a^	4 (5.5)	2 (2.8)
APACHE II at ICU admission, mean (SD)	21.9 (6.8)	20.9 (7.6)
Endotracheal tube size (Fr), *n* (%)		
6.5	1 (1.4)	2 (2.7)
7.0	34 (47.2)	27 (37)
7.5	37 (51.4)	43 (58.9)
8.0	0 (0)	1 (1.4)
Length of intubation, median (IQR), d	6.1 (4.1–10.1)	5.8 (4.2–10.2)
Intubated ≥ 6 d, *n* (%)	37 (51.4)	33 (45.2)
Rapid shallow breathing index, means (SD)	53.1 (26.8)	48.7 (25.7)
> 105, *n* (%)	2 (2.8)	4 (5.5)
*Postextubation baseline*		
GCS level, median (IQR)	15 (14–15)	15 (14–15)
Oxygen demand, *n* (%)		
Nasal cannula	4 (5.6)	5 (6.9)
Simple mask	61 (84.7)	60 (82.2)
NRM or NPPV	7 (9.7)	8 (10.9)
Able to identify 3 shapes lollipop-style test pieces (i.e., Intact Oral stereognosis), *n* (%)^b^	23 (31.9)	15 (20.5)
Cough during 0.4 mol/L citric acid trials, *n* (%)	51 (70.8)	46 (63.0)
Nothing by mouth (FOIS level 1) on the day of extubation, *n* (%)	67 (93.1)	69 (94.5)
Dry mouth, *n* (%)^c^	33/69 (47.8)	29/71 (40.8)

*ICU* intensive care unit; *APACHE II* Acute Physiological and Chronic Health Evaluation II; *IQR* interquartile range; *GCS* Glasgow Coma Scale; *NRM* nonrebreathing mask; *NPPV* noninvasive positive pressure ventilation; *FOIS* Functional Oral Intake Scale

^a^Includes diabetic ketoacidosis and empyema post-surgery

^b^3 shapes of lollipop-style test pieces, including square, star, and round lollipop shapes

^c^Dry mouth was defined as salivary flow ≤ 3 cm/5 min using the oral Schirmer’s test

### Effect on oral feeding

**Fig. 2:**
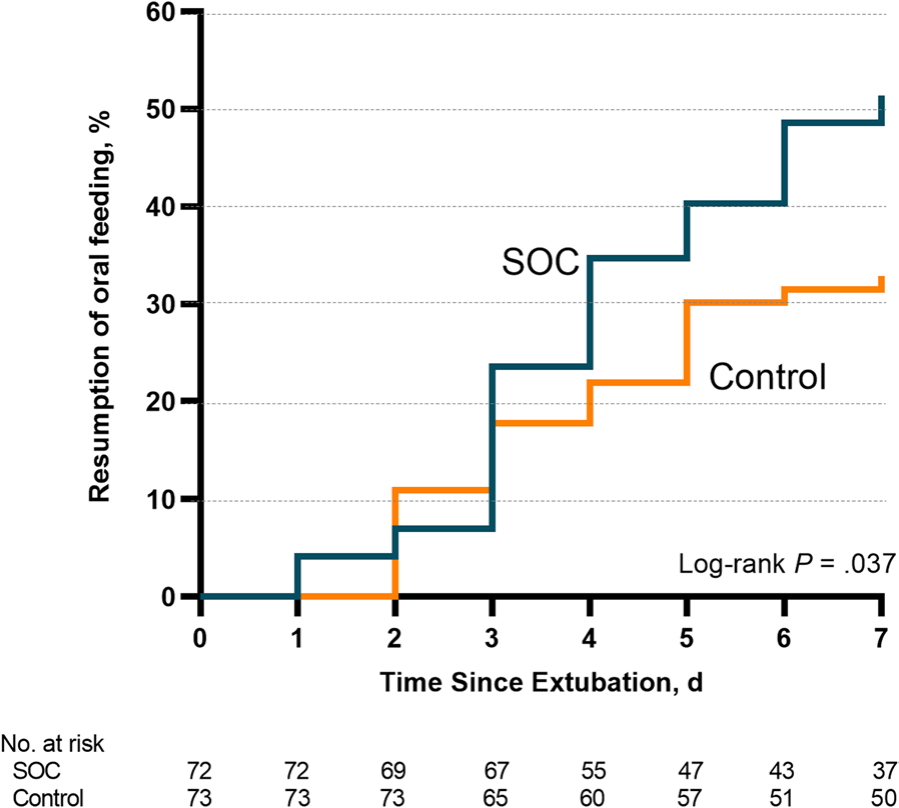
Kaplan–Meier analysis of time from extubation to resumption of oral feeding

A resumption of oral feeding 7-day postextubation occurred in 37/72 participants (51.4%) in the SOC group and in 24/73 participants (32.9%) in the controls (difference, 18.5%; 95% CI 2.7%–34.3%; *P* = 0.024) (Fig. 2). The two groups were similar regarding the median times for the resumption of oral feeding: the median (IQR) was 7 (4.0–7.0) days for the SOC group and 7 (5.0–7.0) days for the controls. We adjusted our analysis of the data for age and intubation longer than 6 days in the Cox regression model and found that participants who had received SOC program had a 2.35-fold higher likelihood of resuming oral feeding by 7 days (adjusted HR, 2.35; 95% CI 1.38–4.01; *P* = 0.0015) than was the case with the control group (Table 2).

**Table 2:** Intervention outcomes (*N* = 145)

Variables	SOC *n* = 72	Control *n* = 73	Group difference* (95% CI)	Adjusted effect estimate^§^ (95% CI)	Adjusted *P* value
Resumption of oral feeding, *n* (%)^a^	37 (51.4)	24 (32.9)	18.5% (2.7, 34.3)	HR, 2.35 (1.38, 4.01)	0.0015
Pneumonia, *n* (%)^b^	11 (15.3)	26 (35.6)	− 20.3% (− 34.1, − 6.5)	OR, 0.28 (0.12, 0.65)	0.0031

*95% CI* 95% confidence interval

*Group difference is the SOC-group value minus the control-group value

^§^Adjusted for age and for intubations longer than 6 days

^a^Hazard ratio according to the Cox proportional-hazards model

^b^Odds ratio according to the logistic regression model (the control group was the reference)

### Effect on pneumonia

Within 30 days following extubation, two participants developed new radiographically documented infiltrates, while 35 had progressive infiltrates during the same studied period, making the overall pneumonia incidence of 25.5% (*n* = 37). Separately, incidence of pneumonia 30 days postextubation was 15.3% (*n* = 11) in the SOC group and 35.6% (*n* = 26) in the controls (difference, − 20.3% [95% CI −34.1 to − 6.5%]; *P* = 0.006). Participants receiving the SOC had reduced odds of developing pneumonia by 72% (adjusted OR = 0.28; 95% CI 0.12–0.65; *P* = 0.0031) than the controls, independent of age and intubated for 6 days and over (Table 2).

### Delivery and adherence of SOC program

The mean number of days that participants in the SOC group received their SOC treatment was 6.2, with an SD of 1.3. Of the 72 participants in the SOC group, 48 (66.7%) received the maximal 7 days of SOC treatment, while one-third (*n* = 24; 33.3%) did not receive a full 7-day intervention owing to discharge from the hospital (*n* = 7), unstable condition (*n* = 3), undergoing a surgical procedure (*n* = 7), or patient refusal due to discomfort or fatigue (*n* = 7). On average, carrying out the three SOC protocols took a mean (SD) of 14.8 (5.7) minutes daily. No adverse events (i.e., coughing, oxygen desaturation [SpO2 < 90%] or respiratory rate > 30 breaths per minute) were reported during or immediately after the intervention.

On the three-point Likert-type adherence scale, adherence to the SOC was satisfactory with a mean score (SD) of 1.77 (0.27). The mean (SD) adherence score for each protocol was 1.83 (0.36) in oral motor exercises, 1.85 (0.26) in sensory stimulation and lubrication, and 1.52 (0.58) in safe-swallowing education. The relatively low score for the safe-swallowing education protocol reflects its scoring rules: the SOC nurse had to determine whether the patients or their caregivers had followed the instructions; full adherence (a score of 2) was rated if a patient sat up during oral intake, did not feed during drowsy states, and modified food texture when needed.

### Sensitivity analysis—whether days of intervention matter

We noticed a shorter-than-expected intervention period, as one-third (*n* = 24; 33.3%) of SOC participants did not receive the complete 7-day intervention for various reasons. Whether “each single intervention day” matters requiring investigation. We used “number of actual SOC-treatment days” (max 7 for the intervention group; 0 for the controls) as an independent variable. As shown in Additional file 3: Table S2, for each SOC day added, odds of resuming oral feeding increased 12% (adjusted HR, 1.12; 95% CI 1.04–1.22; *P* = 0.0045). As to the development of pneumonia, for each SOC day added, the chance of developing pneumonia declined by 17% (adjusted OR, 0.83; 95% CI 0.73–0.95; *P* = 0.006). The more days a patient received SOC program, the more improvement the patient would exhibit. Moreover, the results largely confirm the findings in our main analysis.

### Discussion

SOC program has three central functions: keep patients’ lips, tongue, and jaw moving freely; keep patients’ oral cavity moist, clean, and sensitized with thermo, chemical, and mechanical stimuli; and ensure that patients be well-informed on safe-swallowing strategies. Our RCT demonstrates the positive effects of SOC in critically ill patients receiving prolonged endotracheal intubation. Participants who received daily 14.8-min, nurse-administered SOC program for 6.2 days were 2.35-fold more likely to resume oral feeding than were the participants who received only usual care. More importantly, we found that the 30-day incidence of radiographically documented pneumonia was 72% lower in the SOC group than in the controls.

Previous research has demonstrated that sensorial stimuli (i.e., tactile, chemical, and thermal stimuli) can increase sensorial input to the swallowing center of the brain stem, thus triggering the swallow response earlier and activating sensorimotor integration processes [29–31]. We used locally available food items (e.g., chewy pork jerk and sour-flavor ice pops) to activate sensory receptors located at the soft palate, palatine arch, and posterior part of the tongue [30, 32]. We also offered toothbrushing and saliva gland massage to increase patients’ saliva flow, which in turn provided lubrication necessary for smooth bolus formation and transport of food and liquid during swallowing [33, 34]. These SOC protocols seem commonsensical, yet the key to their effectiveness may lie in their consistent daily application to a recovering patient group that is dysphagia-naïve prior to endotracheal intubation. Similarly, a South Korean RCT study (*n* = 33) applied oropharyngeal exercises and thermal-tactile stimulation to patients under prolonged endotracheal intubation, which showed improvements in the patients’ oropharyngeal swallowing efficiency [35]. Another RCT, conducted in Brazil (*n* = 32), also demonstrated the benefits of a “speech therapy program” for extubated patients’ oral intake [36]. Notably, both studies involved SLPs to provide interventions.

Medical guidelines identify SLPs as key members of the critical-care community in the management of dysphagia [37]. However, many ICUs have no dedicated SLPs. An international survey involving 746 ICUs from 26 countries reported that only 4% of the ICUs had a dedicated SLP [38]. Our study has shown that the nurse-administered SOC adoption can improve functional-swallow outcomes (i.e., the resumption of oral feeding) and pulmonary health (i.e., 30-day pneumonia incidence) in critically ill patients receiving prolonged endotracheal intubation. Medical centers that have not yet have established dysphagia programs may consider SOC a useful starting point to advance point-of-care for these vulnerable ICU patients. For centers that already have an implemented SLP-led dysphagia program, SOC may provide a novel, feasible, structured set of postextubation care protocols that target oral-facial exercise, sensory stimulation, saliva lubrication, and patient education to augment the existing program and enhance recovery.

### Limitations

This study has limitations. *Firstly*, although physicians and staff at the study sites were blinded to the group allocation. The possibility that unintentional unblinding could have occurred when they observed SOC nurses performing detailed swallowing interventions on a number of patients. *Secondly*, only two-thirds of participants received the maximum package of the SOC program. This incomplete delivery is an important limitation. *Thirdly*, the diagnosis of pneumonia has inherent complexities, especially when retrospectively coding from the medical records, which may result in incomplete information in drawing solid conclusions. Future studies might want to verify our findings using a more robust algorithm for pneumonia diagnosis. Moreover, we did not applied FEES to the participants which limit our ability to evaluate the actual dysphagia spectrum in the patients. *Fourthly*, we excluded patients with preexisting dysphagia through the chart review and history taking; this method alone may have limitations, and a more precise exclusion of those with preexisting dysphagia would have been helpful. *Lastly*, the current study design does not allow us to assess the individual contributions of the three SOC components. Future studies could use a step-wedge approach, with each component introduced sequentially, to not only reduce the complexity of introducing the entire intervention package at once, but also to clarify the individual contributions of each component.

## Conclusion

Among critically ill patients receiving a prolonged endotracheal intubation, a daily 14.8-min, nurse-administered, postextubation SOC program significantly improved patients’ return to oral feeding and reduced their incidence of pneumonia. The key to the effectiveness of SOC program is the consistent and daily application of SOC protocols. Medical centers that want to facilitate their patients’ post-ICU recovery might consider SOC an effective starting point for dysphagia and pneumonia prevention.

## Supplementary Information

**Additional file 1.** Swallowing and Oral Care (SOC) Program.

**Additional file 2. Table S1.** Baseline demographics and clinical characteristics between completed and incompleted groups.

**Additional file 3. Table S2.** Length of SOC program relative to the odds of resuming oral feeding and developing pneumonia.

## Data Availability

The datasets used and/or analyzed during the current study are available from the corresponding author on reasonable request.

## Abbreviations

ICU: Intensive-care unit
SOC: Swallowing and oral-care
FOIS: Functional oral intake scale
RCT: Randomized controlled trial
SLP: Speech-language pathologist
APACHE II: Acute Physiological and Chronic Health Evaluation II
NRM: Nonrebreathing mask
NPPV: Noninvasive positive pressure ventilation
IQR: Interquartile ranges
CI: Confidence interval
ORs: Odds ratios
