# Supplementary material for: Effects of a swallowing and oral-care program on resuming oral feeding and reducing pneumonia in patients following endotracheal extubation: a randomized, open-label, controlled trial
Source: Crit Care. 2023 Jul 12;27:283. doi: 10.1186/s13054-023-04568-6 (PMC10339550; doi:10.1186/s13054-023-04568-6)
Supplement: Supplementary file 1 — Additional file 1. Swallowing and Oral Care (SOC) Program. [file 13054_2023_4568_MOESM1_ESM.pdf]

Swallowing and Oral Care (SOC) Program

Oral motor exercise of lips, tongue, jaw, and cheeks

1. Active-assistive range of motion (ROM)
    - Patients are asked to purse their lips and stretch them to one side of the face and then to the other; patients extend their tongue out of the mouth in forward, right, and left directions before retracting the tongue back into the mouth; finally, patients open their mouths widely. Depending on their tolerance, patients perform up to 10 repetitions of these steps.
2. Resistive ROM when tolerated
    - Patients are asked to push their lips outward against the opposing force of a tongue depressor; patients then extend their tongue out of the mouth in a forward direction against the opposing force of a tongue depressor; participants open their mouth to the maximum degree against an opposing force created by the SOC nurse's tongue depressor. Depending on their tolerance, patients perform up to 10 repetitions of these steps.

Sensory stimulation and lubrication

- The SOC nurse places a sour-flavor ice pop into a patient's mouth and asks the patient to lick and suck it until the ice pop has disappeared; the SOC nurse places a chewy jerky into a patient's mouth and asks the patient to chew it thoroughly.
- The SOC nurse rinses the patient's oral cavity with distilled water to soften sticky coated plaque; brushes the patient's teeth, gums, tongue, and palate with a soft toothbrush; wipes the plaque off with a wet gauze; and then places between 1 and 2 cm of Biotene® gel-based saliva substitute directly onto the lips and throughout the inside of the mouth, including the tongue and oral mucosa.
- The SOC nurse gently massages the edge of the patient's cheek area in front of the ears and then moves the massage along the chin until both the area under the jaw and the tongue area are reached; finally, with two fingers, the nurse gently presses 5 to 10 times in the submental area to "milk" the parotid gland, the submandibular gland, and the sublingual gland.

Safe-swallowing education

- The SOC nurse explains to patients the signs and symptoms of unsafe swallowing.
- The SOC nurse advises patients on safe-swallowing strategies, including sitting up during oral intake, no feeding in a drowsy or sleepy state, and modifying dietary texture and viscosity.
