# Supplementary material for: Effects of a swallowing and oral-care program on resuming oral feeding and reducing pneumonia in patients following endotracheal extubation: a randomized, open-label, controlled trial
Source: Crit Care. 2023 Jul 12;27:283. doi: 10.1186/s13054-023-04568-6 (PMC10339550; doi:10.1186/s13054-023-04568-6)
Supplement: Supplementary file 2 — Additional file 2. Table S1. Baseline demographics and clinical characteristics between completed and incompleted groups. [file 13054_2023_4568_MOESM2_ESM.pdf]

Supplemental Table 1 Baseline demographics and clinical characteristics between completed and incompleted groups

**Variables**	**Complete** (n=117)	**Incomplete** (n=28)	**P-value**
Age, mean (SD), y	65.7 (15.0)	66.3 (14.9)	1.00
Age ≥ 70 y, n (%)	50 (42.7)	13 (46.4)	.723
Male, n (%)	72 (61.)	13 (45.4)	.144
Body mass index, mean (SD)	23.5 (4.4)	23.0 (4.0)	.579
Charlson Comorbidity Index, mean (SD)	2.6 (2.3)	3.5 (2.8)	.077
ICU admission diagnosis, n (%)			.088
Respiratory failure	64 (54.7)	20 (71.4)	
Cardiac emergency	26 (22.2)	1 (3.6)	
Noncardiogenic shock	21 (18.0)	7 (25.0)	
Others[a]	6 (4.3)	0 (0.0)	
APACHE II at ICU admission, mean (SD)	21.6 (7.4)	20.8 (5.9)	.609
Endotracheal tube size (Fr), n (%)			.929
6.5	3 (2.6)	0 (0.0)	
7.0	48 (41.0)	13 (46.4)	
7.5	65 (55.6)	15 (53.6)	
8.0	1 (0.9)	0 (0.0)	
Length of intubation, median (IQR), d	5.4 (3.9-9.8)	8.8 (6.3-14.8)	.0007
Intubated ≥ 6 d, n (%)	48 (41.0)	22 (78.6)	.0004
Rapid shallow breathing index, means (SD)	47.8 (25.9)	64.1 (23.7)	.0034
> 105, n (%)	4 (3.4)	2 (7.1)	.327
**Postextubation baseline**			
GCS level, median (IQR)	15 (15-15)	15 (13.5-15)	.108
Oxygen demand, n (%)			.295
Nasal cannula	6 (5.1)	3 (10.7)	
Simple mask	100 (85.5)	21 (75.0)	
NRM or NPPV	11 (9.4)	4 (14.3)	
Able to identify 3 shapes of lollipop-style test pieces (i.e. Intact Oral stereognosis), n (%)[b]	28 (23.9)	10 (35.7)	.202
Able to cough during 0.4 mol/L citric acid inhalation trials, n (%)	77 (65.8)	20 (71.4)	.658
Nothing by mouth (FOIS level 1) on the day of extubation, n (%)	108 (92.3)	28 (100)	.574
Dry mouth, n (%)[c]	49/117 (41.8)	13/28 (46.4)	.662

Abbreviations: ICU, intensive care unit; APACHE II, Acute Physiological and Chronic Health Evaluation II; IQR, interquartile range; GCS, Glasgow Coma Scale; NRM, nonrebreathing mask; NPPV, noninvasive positive pressure ventilation; FOIS, Functional Oral Intake Scale.

[a]Includes diabetic ketoacidosis and empyema post-surgery.

[b]Three lollipop-style oral test pieces including square, star, and round lollipop shapes

[c]Dry mouth was defined as salivary flow ≤ 3 cm/5 min using using the oral Schirmer's test
