# Supplementary material for: Effects of a swallowing and oral-care program on resuming oral feeding and reducing pneumonia in patients following endotracheal extubation: a randomized, open-label, controlled trial
Source: Crit Care. 2023 Jul 12;27:283. doi: 10.1186/s13054-023-04568-6 (PMC10339550; doi:10.1186/s13054-023-04568-6)
Supplement: Supplementary file 3 — Additional file 3. Table S2. Length of SOC program relative to the odds of resuming oral feeding and developing pneumonia. [file 13054_2023_4568_MOESM3_ESM.pdf]

Supplemental Table 2 Length of SOC program relative to the odds of resuming oral feeding and developing pneumonia

Variables	Resumption of oral feeding[a]		Pneumonia[b]	
	**Adjusted effect estimate[§] (95% CI)**	**Adjusted P-value**	**Adjusted effect estimate[§] (95% CI)**	**Adjusted P-value**
Days receiving SOC program	HR, 1.12 (1.04, 1.22)	.0045	OR, 0.83 (0.73, 0.95)	.006

Abbreviations: 95% CI, 95% confidence interval

[§]Adjusted for age and intubations longer than 6 days.

[a]Hazard ratio according to the Cox proportional-hazards model.

[b]Odds ratio according to the logistic regression model (the control group was the reference).
